# Distinct genetic clades of enterovirus D68 detected in 2010, 2013, and 2015 in Osaka City, Japan

**DOI:** 10.1371/journal.pone.0184335

**Published:** 2017-09-13

**Authors:** Atsushi Kaida, Nobuhiro Iritani, Seiji P. Yamamoto, Daiki Kanbayashi, Yuki Hirai, Masao Togawa, Kiyoko Amo, Urara Kohdera, Toshinori Nishigaki, Masashi Shiomi, Sadasaburo Asai, Tsutomu Kageyama, Hideyuki Kubo

**Affiliations:** 1 Division of Microbiology, Osaka Institute of Public Health, Osaka, Japan; 2 Osaka City General Hospital, Osaka, Japan; 3 Nakano Children’s Hospital, Osaka, Japan; 4 Osaka Police Hospital, Osaka, Japan; 5 Aizenbashi Hospital, Osaka, Japan; 6 Asai Children’s Clinic, Osaka, Japan; 7 Influenza Virus Research Center, National Institute of Infectious Diseases, Tokyo, Japan; University of Malaya, MALAYSIA

## Abstract

The first upsurge of enterovirus D68 (EV-D68), a causative agent of acute respiratory infections (ARIs), in Japan was reported in Osaka City in 2010. In this study, which began in 2010, we surveyed EV-D68 in children with ARIs and analyzed sequences of EV-D68 strains detected. Real-time PCR of 19 respiratory viruses or subtypes of viruses, including enterovirus, was performed on 2,215 specimens from ARI patients (<10 years of age) collected between November 2010 and December 2015 in Osaka City, Japan. EV-D68 was identified in 18 enterovirus-positive specimens (*n* = 4 in 2013, *n* = 1 in 2014, and *n* = 13 in 2015) by analysis of viral protein 1 (VP1) or VP4 sequences, followed by a BLAST search for similar sequences. All EV-D68 strains were detected between June and October (summer to autumn), except for one strain detected in 2014. A phylogenetic analysis of available VP1 sequences revealed that the Osaka strains detected in 2010, 2013, and 2015 belonged to distinct clusters (Clades C, A, and B [Subclade B3], respectively). Comparison of the 5′ untranslated regions of these viruses showed that Osaka strains in Clades A, B (Subclade B3), and C commonly had deletions at nucleotide positions 681–703 corresponding to the prototype Fermon strain. Clades B and C had deletions from nucleotide positions 713–724. Since the EV-D68 epidemic in 2010, EV-D68 re-emerged in Osaka City, Japan, in 2013 and 2015. Results of this study indicate that distinct clades of EV-D68 contributed to re-emergences of this virus in 2010, 2013, and 2015 in this limited region.

## Introduction

Enterovirus D68 (EV-D68) is a single-stranded, positive-sense RNA virus that belongs to the family *Picornaviridae*, genus *Enterovirus*, and species *Enterovirus D* [1]. EV-D68 was first isolated in the US in 1962 from four children with pneumonia and bronchitis [2]. EV-D68 was then rarely reported until the early 2000s. In fact, only 26 cases were reported in the US from 1975 to 2005 [3]. Around 2010, epidemics of EV-D68 coincident with acute respiratory infections (ARIs) were reported worldwide [4]. EV-D68 was also detected in patients with acute flaccid paralysis (AFP)/acute flaccid myelitis (AFM) in the US, Norway, and France from 2012 to 2014 [5–9].

The EV-D68 genome encodes a precursor polyprotein between the 5′- and 3′-untranslated regions (UTRs). After proteolytic processing, the following mature proteins are synthesized: structural proteins (viral protein [VP] 4, VP2, VP3, and VP1) and non-structural proteins (2A, 2B, 2C, 3A, 3B, 3C, and 3D). The surface of the viral capsid is composed mainly of VP1, VP2, and VP3, and the nucleotide sequence of VP1 has been used for phylogenetic analyses because it corresponds with virus antigenicity [10].

Recently detected EV-D68 strains belong to four major genetic clades (A, B, C, and D), as determined by molecular analyses of the VP1 gene [11–14]. Clade B has three subclades (B1, B2, and B3) [11, 15, 16]

The first reported EV-D68 upsurge in Japan occurred in Osaka City, the second largest city in Japan with 2.7 million residents, in 2010; a single genetic clade was responsible (Clade C) [17]. In this study, we surveyed EV-D68 in children with ARIs since that first epidemic in 2010 and molecularly analyzed the strains detected.

This is the first report of longitudinal surveillance of EV-D68 in children with ARIs in a limited geographic area in Japan since the 2010 epidemic. Here, re-emergence of EV-D68 was observed in patients with ARIs mainly in 2013 and 2015, with distinct genetic clades responsible for epidemics in each of these years.

## Materials and methods

### Clinical specimens

From November 2010 to December 2015, 1,905 respiratory specimens were obtained by pediatricians from children (<10 years of age) suspected of having viral ARIs in a passive surveillance program (with influenza-diagnosed specimens were excluded) conducted in Osaka City, Japan [18]. In addition, 310 respiratory specimens were collected from hospitals and clinics from April to December 2015 for cooperative research. Surveillance in Osaka City was part of a national surveillance program for viral infectious diseases in Japan based on the Infectious Diseases Control Law. Specimens, collected as part of a passive surveillance, were residues after clinical use in hospitals and clinics. Specimens were anonymized before testing in our public health laboratory. Sample collection, detection of pathogens, and analysis of detected pathogens were approved by domestic law without additional permissions. Specifically, informed consent is considered unnecessary by domestic law and according to ethical guidelines for epidemiological research. However, sample collection for the cooperative research, which was not considered virus surveillance, required both informed consent and ethics committee review. Therefore, written consent was obtained from parents and guardians of the children whose specimens were included in the study. This part of the study was approved by the Ethics Committee of the National Institute of Infectious Diseases, Tokyo, Japan (no. 532).

### EV-D68 identification

Viral nucleic acid was extracted using a QIAamp Viral RNA Mini Kit (Qiagen Inc., Hilden, Germany). Then, cDNA was synthesized using SuperScript III (Thermo Fisher Scientific Inc., Waltham, MA, USA) or PrimeScript Reverse Transcriptase (Takara Bio Inc., Shiga, Japan) with random hexamer primers. Multiplex real-time PCR was performed using a kit (QuantiTect Multiplex PCR; Qiagen Inc.) to detect 19 viruses or subtypes of viruses, including human metapneumovirus, respiratory syncytial virus (A, B), human parainfluenza virus types 1–4, human bocavirus, human coronavirus (229E, OC43, HKU1, NL63), influenza virus (A [FLUAV], A H1N1 2009 [FLUA (H1N1) 2009], B [FLUBV], C [FLUCV]), human adenovirus, human enterovirus (HEV), and human rhinovirus, as described previously [18]. For enterovirus-positive specimens, the VP1 or VP4 region was amplified by conventional reverse transcription-PCR. VP1 was amplified by PCR using primers 224 (5′-GCIATGYTIGGIACICAYRT-3′) and 222 (5′-CICCIGGIGGIAYRWACAT-3′), followed by a nested PCR using primers AN89 (5′-CCAGCACTGACAGCAGYNGARAYNGG-3′) and AN88 (5′-TACTGGACCACCTGGNGGNAYRWACAT-3′) [19]. VP4 was amplified using primers OL68-1 (5′-GGTAAYTTCCACCACCANCC-3′) and EVP4 (5′-CTACTTTGGGTGTCCGTGTT-3′) [20]. PCR products were subjected to direct sequencing, and EV-D68 was identified by phylogenetic analysis.

### EV-D68 genome sequence determination

The complete or nearly complete genome sequences of EV-D68 strains from Osaka in 2013 and 2015 were determined as follows: cDNA was synthesized using primers (random hexamer or sequence-specific) and SuperScript III; then, EV-D68 genomes were amplified in nine fragments using the primers shown in S1 Table. PCR was performed using KOD-plus Neo DNA Polymerase (TOYOBO, Tokyo, Japan) with the following conditions: 94°C for 2 min, followed by 35–45 cycles at 98°C for 10 s, 55°C for 30 s, and 68°C between 30 s and 4 min according to the manufacturer’s instructions. PCR products were purified using a QIAquick PCR Purification Kit (Qiagen Inc.). End-specific nucleotide sequences were determined using the 5′ RACE system (Thermo Fisher Scientific) and 3′ RACE with primer TX30SXN [21]. Nucleotide sequences were determined using an ABI 3130 Genetic Analyzer (Thermo Fisher Scientific).

### Molecular analysis

Available nucleotide sequences were obtained from GenBank. The nucleotide sequences of the 14 EV-D68 strains identified in this study were deposited into the DDBJ/EMBL/GenBank database (accession nos. LC068709–LC068711, LC107890–LC107897, LC107899–LC107901). Complete or near-complete genome sequences of 58 strains of EV-D68 obtained worldwide were used for the phylogenetic analyses. In addition, sequences of VP1 (927 nt, corresponding to nucleotide positions 2,389–3,315 of the Fermon strain) from 110 strains were used to construct the phylogenetic tree. Sequences were aligned in MUSCLE, and the phylogenetic tree was constructed using the maximum likelihood method with 1,000 bootstrap replicates in MEGA 7.0 [22]. Genetic clades were applied based on a previous report [11].

To analyze the 5′ UTR sequences of the EV-D68 strains, sequences at nucleotide positions 501–1000 were aligned in MEGA 7.0 and visualized using Genetyx ver. 13 (Genetyx Corp., Tokyo, Japan).

## Results

### Re-emergences of EV-D68 in children with ARIs in 2013, 2014, and 2015

**Fig 1:**
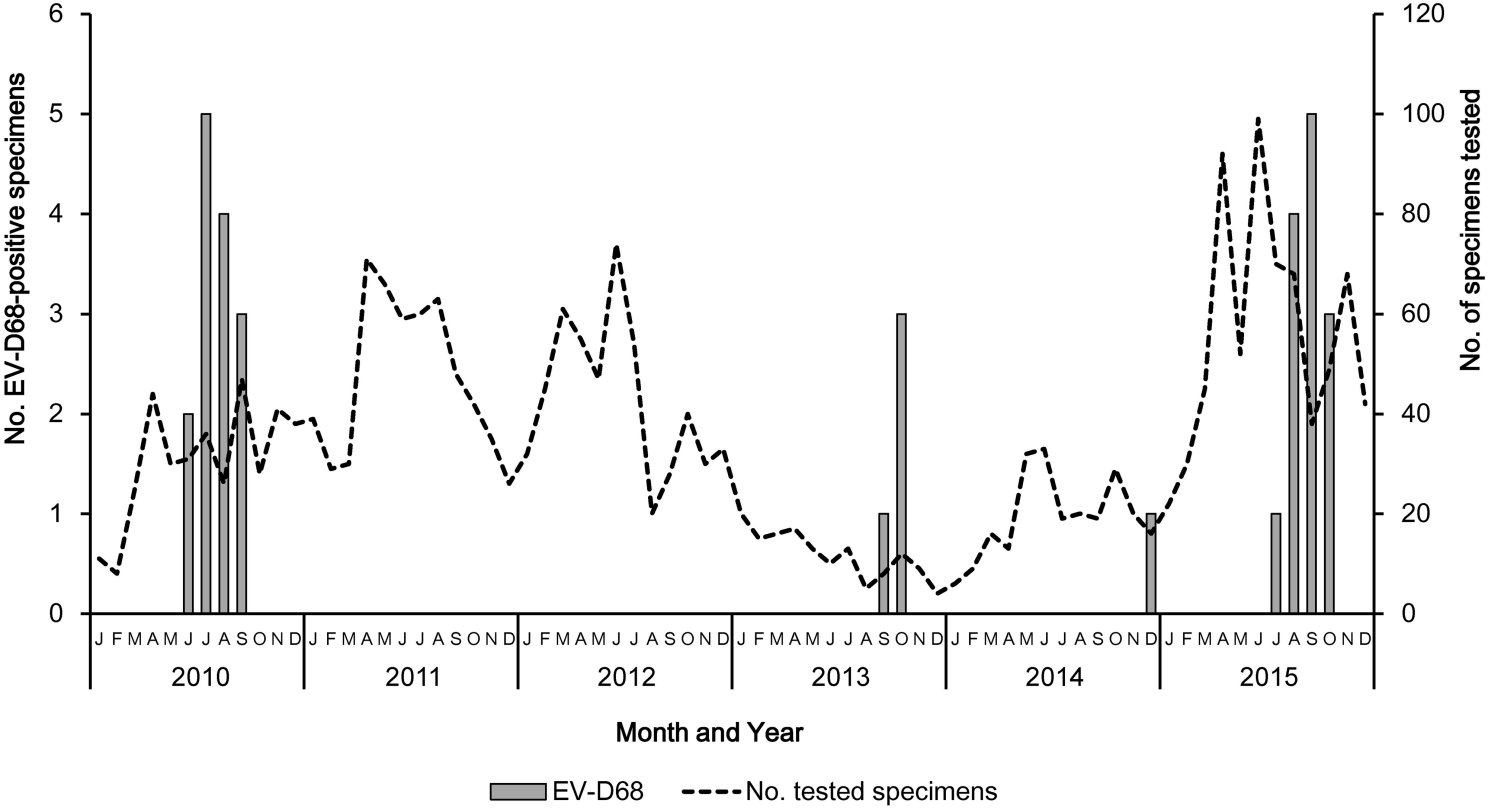
Distribution of EV-D68 in Osaka City, Japan, from January 2010 to December 2015. The unbroken line represents the number of specimens collected per month. The shaded bar shows EV-D68-positive specimens from patients with ARIs. Our previous data from January to October 2010 are also shown.

The monthly distribution of EV-D68 from 2010 to 2015 is presented in Fig 1. Our previously reported data on EV-D68 strains detected in patients with ARI between January and October 2010 [17] were also added to the figure. After the upsurge of EV-D68 in 2010, no EV-D68 was detected in 2011 or 2012. However, re-emergences of EV-D68 were observed in 2013, 2014, and 2015. In all, 18 EV-D68 strains were detected (*n* = 4, 2.8% in 2013; *n* = 1, 0.4% in 2014; and *n* = 13, 1.9% in 2015). To exclude the effects of different sample sizes collected each month, the rate of EV-D68 positivity per month was calculated: 6.5% (June), 13.9% (July), 15.4% (August), and 6.4% (September) in 2010; 12.5% (September) and 25% (October) in 2013; 6.3% (December) in 2014; 1.4% (July), 5.9% (August), 13.2% (September), and 6.1% (October) in 2015. EV-D68 was detected from June to October (summer to autumn) in 2010, 2013, and 2015, but not in 2014.

The 18 EV-D68-positive patients (9 males, 9 females) (nos. 1–18 in Table 1) belonged to different age groups: 0–11 months (*n* = 6, 33.3%), 1 year (*n* = 4, 22.2%), 2 years (*n* = 1, 5.6%), 3 years (*n* = 3, 22.2%), 4 years (*n* = 2, 11.1%), 6 years (*n* = 1, 5.6%), and 9 years (*n* = 1, 5.6%). Patients <2 years of age accounted for 55.6% of all EV-D68-positive cases in the study. Almost all patients showed signs of lower respiratory tract infections and were diagnosed with the following: pneumonia (*n* = 6), asthmatic bronchitis (*n* = 5), asthma (*n* = 2), bronchial asthma (*n* = 2), bronchitis (*n* = 1), respiratory failure (*n* = 1), and pertussis or viral ARI (suspected) (*n* = 1). Of the 18 EV-D68-positive patients, 15 (83.3%) were positive for EV-D68 alone. In the remaining three patients, co-infections of EV-D68 with adenovirus or rhinovirus (Table 1) were identified. No associations between genetic clade and clinical signs were observed.

**Table 1:** Information on EV-D68-positive patients and EV-D68 detected in 2010–2015 in Osaka City, Japan.

No.	Name	Specimens	Age	Gender	Diagnosis	Sampling Date	Co-detection	Clade	Sequenced Region	Nucleotides sequenced	GenBank No.
1	705-OsakaC-JPN-2013	Tracheal aspirate	0 y 4 m	M	Viral bronchitis, respiratory failure	2013/9/30	Rhinovirus	A	Complete genome	1–7341	LC068709
2	726-OsakaC-JPN-2013	Nasal mucus	2 y 0 m	M	Asthmatic bronchitis	2013/10/8	Adenovirus	A	Complete genome	1–7341	LC068710
3	727-OsakaC-JPN-2013	Nasal mucus	6 y 0 m	F	Pneumonia	2013/10/4		A	Nearly complete genome	48–7342	LC068711
4	752-OsakaC-JPN-2013	Throat swab	9 y 1 m	M	Asthmatic bronchitis	2013/10/22		ND*	VP4	NR**	NR**
5	1082-OsakaC-JPN-2014	Nasal mucus	1 y 7 m	M	Pneumonia	2014/12/1		ND*	VP4	NR**	NR**
6	594-OsakaC-JPN-2015	Nasal mucus	4 y 1 m	M	Asthma, bronchitis	2015/8/15		B (Subclade B3)	Nearly complete genome	1–7296	LC107895
7	639-OsakaC-JPN-2015	Nasal mucus	3 y 10 m	M	Asthma, bronchitis	2015/8/28		B (Subclade B3)	Nearly complete genome	1–7286	LC107896
8	676-OsakaC-JPN-2015	Nasal mucus	0 y 9 m	M	Asthmatic bronchitis	2015/9/11		ND*	Partial VP1	NR**	NR**
9	712-OsakaC-JPN-2015	Nasal mucus	1 y 6 m	F	Pneumonia	2015/9/20		ND*	Partial VP1	NR**	NR**
10	744-OsakaC-JPN-2015	Nasal mucus	1 y 7 m	F	Asthmatic bronchitis	2015/10/5		B (Subclade B3)	Complete VP1	927	LC107890
11	759-OsakaC-JPN-2015	Nasal mucus	3 y 8 m	F	Asthmatic bronchitis	2015/10/8		B (Subclade B3)	Complete VP1	927	LC107891
12	A184-OsakaC-JPN-2015	Throat swab	0 y 2 m	F	Pertussis or viral ARI (suspected)	2015/7/27		B (Subclade B3)	Nearly complete genome	1–7286	LC107897
13	A194-OsakaC-JPN-2015	Throat swab	3 y 11 m	F	Pneumonia, athma	2015/8/4		B (Subclade B3)	Complete VP1	927	LC107892
14	A216-OsakaC-JPN-2015	Throat swab	4 y 11 m	F	Bronchial asthma	2015/8/18		B (Subclade B3)	Complete VP1	927	LC107893
15	A244-OsakaC-JPN-2015	Nasopharyngeal aspirate	0 y 7 m	F	Pneumonia	2015/9/11	Adenovirus	B (Subclade B3)	Nearly complete genome	1–7298	LC107899
16	A250-OsakaC-JPN-2015	Nasopharyngeal aspirate	1 y 2 m	F	Pneumonia, athma	2015/9/17		B (Subclade B3)	Nearly complete genome	1–7112	LC107900
17	A252-OsakaC-JPN-2015	Nasopharyngeal aspirate	0 y 10 m	M	Bronchitis	2015/9/18		B (Subclade B3)	Nearly complete genome	1–7110	LC107901
18	A274-OsakaC-JPN-2015	Throat swab	0 y 10 m	M	Bronchial asthma	2015/10/19		B (Subclade B3)	Complete VP1	927	LC107894
19	200-OsakaC-JPN-2010	Nasal mucus	4 y	M	Bronchitis	2010/6/4		C	Complete VP1	927	AB601872
20	290-OsakaC-JPN-2010	Nasal mucus	0 y 7 m	F	Asthmatic bronchitis	2010/6/26		C	Complete genome	1–7332	AB601882
21	373-OsakaC-JPN-2010	Nasal mucus	3 y 6 m	M	Bronchitis	2010/7/17		C	Complete VP1	927	AB601873
22	378-OsakaC-JPN-2010	Nasal mucus	1 y 6 m	F	Pneumonia	2010/7/16		C	Complete genome	1–7331	AB601883
23	396-OsakaC-JPN-2010	Nasal mucus	4 y 6 m	F	Pneumonia	2010/7/22		C	Nearly complete genome	1–6764	AB601884
24	402-OsakaC-JPN-2010	Nasal mucus	5 y 0 m	F	Asthma, respiratory failure	2010/7/23		C	Complete VP1	927	AB601874
25	412-OsakaC-JPN-2010	Sputum	3 y 9 m	M	Lower respiratory tract infection	2010/7/23		C	Complete VP1	927	AB601875
26	441-OsakaC-JPN-2010	Nasal mucus	1 y 3 m	F	Asthmatic bronchitis	2010/8/1		C	Complete VP1	927	AB601876
27	445-OsakaC-JPN-2010	Sputum	4 y 1 m	M	Asthmatic bronchitis	2010/8/1		C	Complete VP1	927	AB601877
28	471-OsakaC-JPN-2010	Nasal mucus	1 y 6 m	M	Bronchial pneumonia	2010/8/5		C	Complete VP1	927	AB601878
29	515-OsakaC-JPN-2010	Throat swab	0 y 3 m	M	Upper respiratory tract infection	2010/8/19		C	Partial VP1	NR**	NR**
30	573-OsakaC-JPN-2010	Throat swab	3 y 5 m	M	Asthmatic bronchitis	2010/9/1		C	Complete VP1	927	AB601879
31	616-OsakaC-JPN-2010	Nasal mucus	0 y 7 m	M	Asthmatic bronchitis	2010/9/13	Adenovirus	C	Complete VP1	927	AB601880
32	618-OsakaC-JPN-2010	Nasal mucus	1 y 5 m	M	Pneumonia	2010/9/11		C	Complete VP1	927	AB601881
33	404-OsakaC-JPN-2010	Nasal mucus	1 y 8 m	M	Febrile convulsion	2010/7/25		C	Nearly complete genome	1–6764	AB601885
34	692-OsakaC-JPN-2013	Nasal mucus	0 y 9 m	M	Acute flaccid paralysis, diarrhea	2013/9/24	Human parechovirus 1	A	Complete genome	1–7341	LC068708
35	685-OsakaC-JPN-2015	Throat swab	2 y 2 m	M	Unknown fever	2015/9/15		B (Subclade B3)	Complete VP1	927	LC107889
36	A166-OsakaC-JPN-2015	Throat swab	1 y 10 m	F	Rash	2015/7/10	Human coronavirus OC43	ND*	Partial VP1	NR**	NR**
37	A241-OsakaC-JPN-2015	Throat swab	0 y 2 m	F	Fever	2015/9/7		B (Subclade B3)	Nearly complete genome	1–7196	LC107898

ND*: Not determined

NR**: Not registered

ARI: Acute respiratory infection

No. 1–18: EV-D68 positives with respiratory symptoms in this study.

No. 19–32: EV-D68 positives with respiratory symptoms in our previous study (15).

No. 33–37: EV-D68 positives without respiratory symptoms.

### Phylogenetic analysis of the VP1 gene

**Fig 2:**
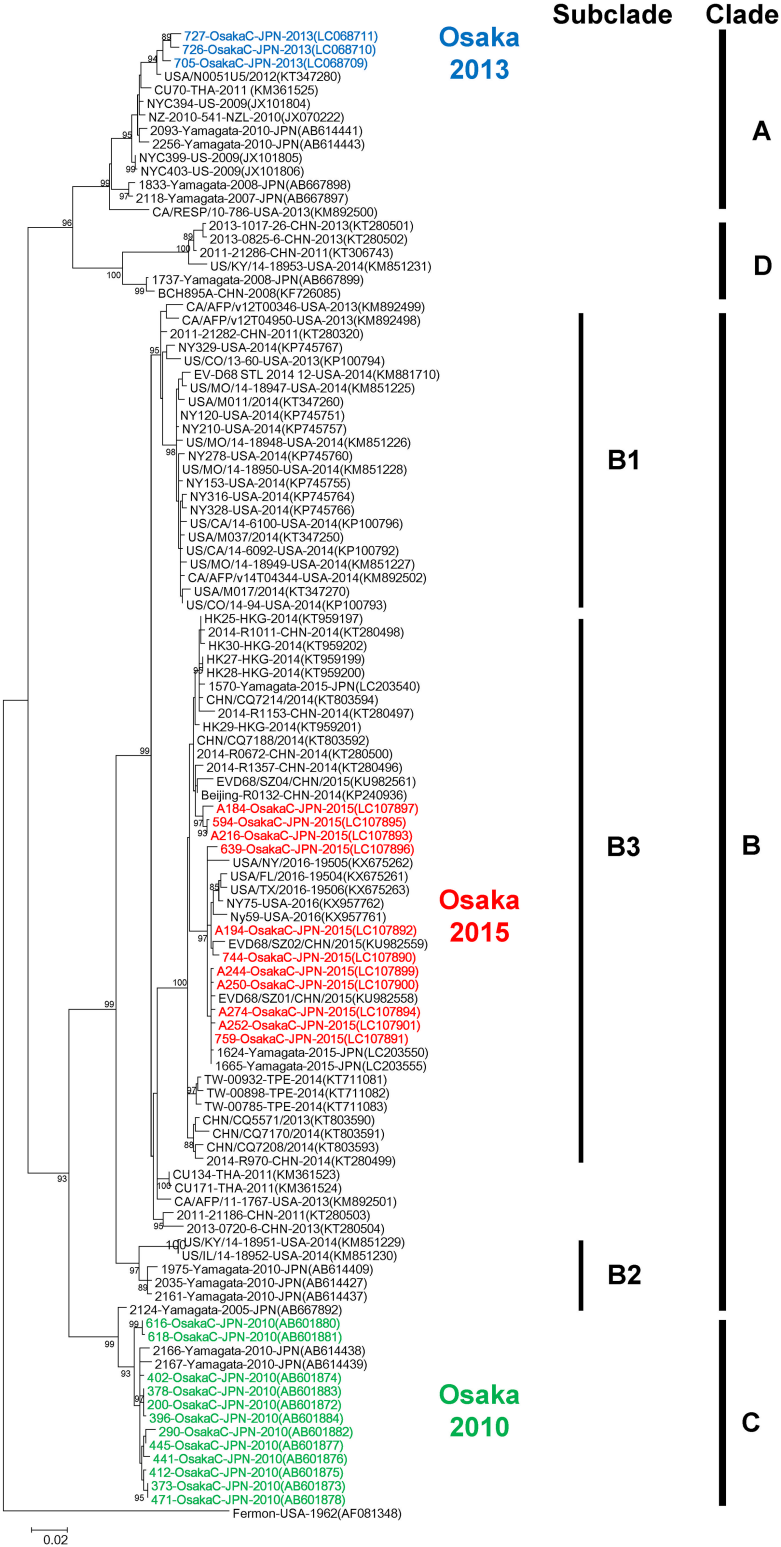
Phylogeny of complete VP1 gene sequences. Complete VP1 gene sequences (927 nt, corresponding to nucleotide positions 2,389–3,315 of the Fermon prototype strain of EV-D68) were analyzed. The phylogenetic tree was constructed and evaluated with 1,000 bootstrap pseudoreplicates using MEGA 7.0 software. Based on the Akaike information criterion with correction for finite sample sizes, a general time reversible (GTR) plus gamma distributed with invariant sites (G+I) model was used. Numbers at nodes, which indicate bootstrap support values (>85%), are shown. Sequences in GenBank were also included in the analysis. Strain name, country of origin, and year of detection are shown for each strain. GenBank accession numbers are presented in parentheses. The scale bar shows the genetic distance.

The phylogenetic analysis was performed using available EV-D68 sequences obtained worldwide and Osaka strains obtained in 2010, 2013, and 2015. One EV-D68 strain detected in 2014 could not be amplified. Osaka strains detected in 2010, 2013, and 2015 belonged to Clade C, Clade A, and Clade B (Subclade B3), respectively (Fig 2). Clade names were assigned according to an earlier report [11].

Nucleotide and amino acid sequence similarities among Osaka strains were the following: 98.2–100% (nt) and 98.7–100% (aa) in 2010; 98.5–99.0% (nt) and 100% (aa) in 2013; 97.3–99.8% (nt) and 98.0–100% (aa) in 2015; 89.3–90.5% (nt) and 94.8–95.4% (aa) between stains obtained in 2010 and 2013; 89.8–91.3% (nt) and 94.4–96.1% (aa) between strains obtained in 2010 and 2015; and 86.8–87.8% (nt) and 94.1–95.1% (aa) between strains obtained in 2013 and 2015. Osaka strains detected in the same year showed high similarities both in nucleotide and amino acid sequences; however, lower similarities were observed between strains detected in different years. When VP1 nucleotide and amino acid sequences of Osaka strains detected in 2015 (Subclade B3) were compared with recently reported strains from United States, China, Hong Kong, Taiwan, and Yamagata, Japan, shown in Fig 2, they were found to be highly similar (96.5–100% [nt]; 97.1–100% [aa]) (Table 2).

**Table 2:** VP1 amino acid and nucleotide sequence identities between subclade B3 strains from Osaka and worldwide.

Country	No. strains	Year of detection	Nucleotide identity (%)	Amino acid identity (%)
United States	5	2016	96.5–99.4	97.7–99.7
Taiwan	3	2014	97.5–98.3	98.1–99.7
Hong Kong	5	2014	97.7–99.0	99.0–100
China	14	2013–2015	97.2–99.8	97.1–100
Yamagata, Japan	3	2015	97.4–100	98.4–100

### Phylogenetic analysis using complete or near-complete genome sequences of EV-D68 strains

**Fig 3:**
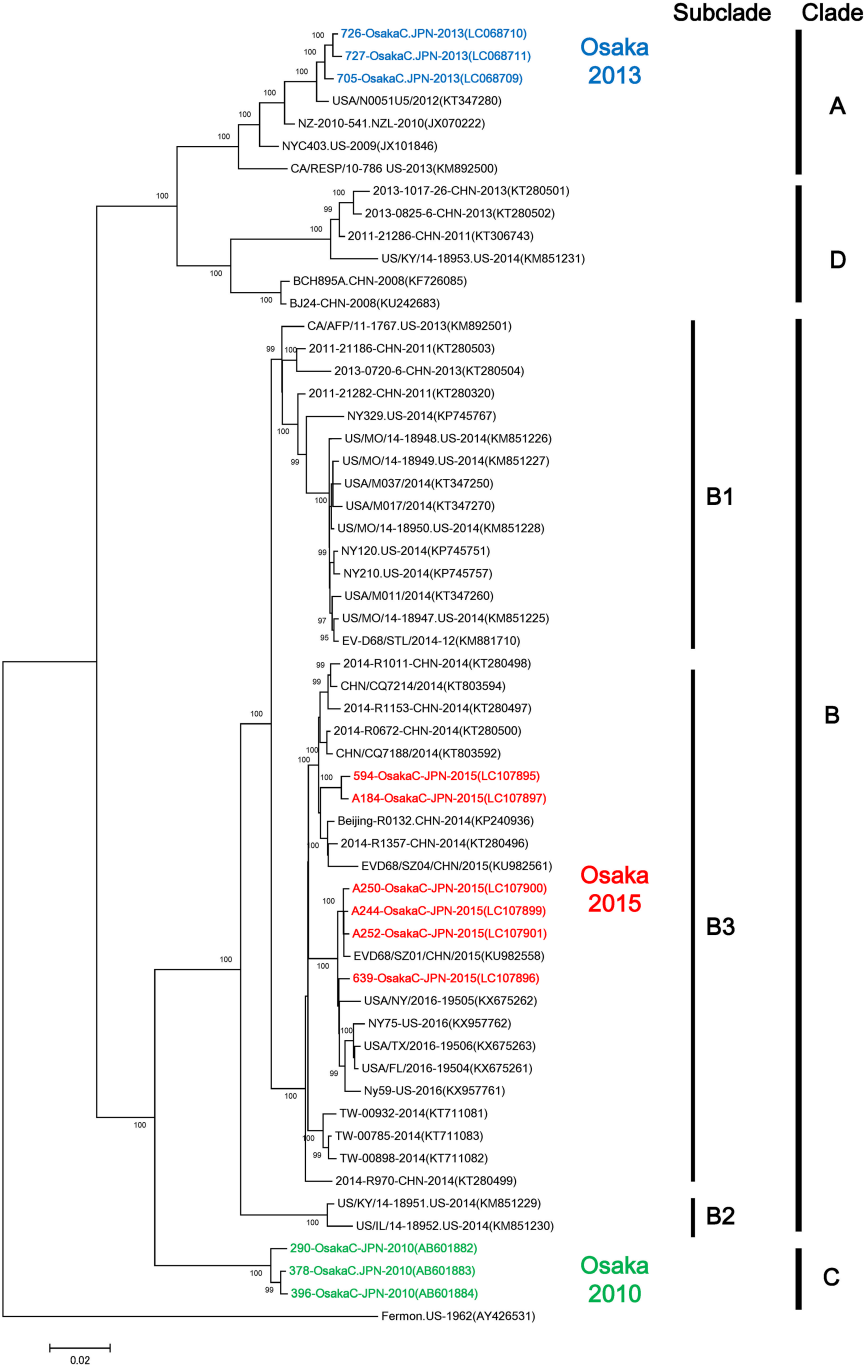
Phylogenetic analysis of complete or near-complete genome sequences of EV-D68 strains. Complete or near-complete genome sequences of EV-D68 were used to construct a maximum likelihood phylogram. The phylogenetic tree was constructed and evaluated with 1,000 bootstrap pseudoreplicates using MEGA 7.0 software. Based on the Akaike information criterion with correction for finite sample sizes, a GTR plus G+I model was used. Numbers at nodes, which indicate bootstrap support values (>95%), are given. Sequences in GenBank were also included in the analysis. Strain name, country of origin, and year of detection are shown for each strain. GenBank accession numbers are presented in parentheses. The scale bar shows the genetic distance.

To analyze and compare the strains detected in this study with strains reported earlier from around the world, complete genome sequences (7,341 nt) of two strains (705-OsakaC.JPN-2013, 726-OsakaC.JPN-2013) and near-complete genome sequences (7,110–7,298 nt) from seven strains (727-OsakaC.JPN-2013, 594-OsakaC.JPN-2015, 639-OsakaC.JPN-2015, A184-OsakaC.JPN-2015, A244-OsakaC.JPN-2015, A250-OsakaC.JPN-2015, A252-OsakaC.JPN-2015) were determined. The phylogenetic analysis of EV-D68 strains from Osaka showed that these strains belonged to three distinct clades (A, B, and C). Specifically, Osaka strains detected in 2010, 2013, and 2015 clustered into Clades C, A, and B (Subclade B3), respectively (Fig 3). This genogrouping was similar to that based on the VP1 analysis (Fig 2).

### Amino acid alignment of the partial VP1 region

**Fig 4:**
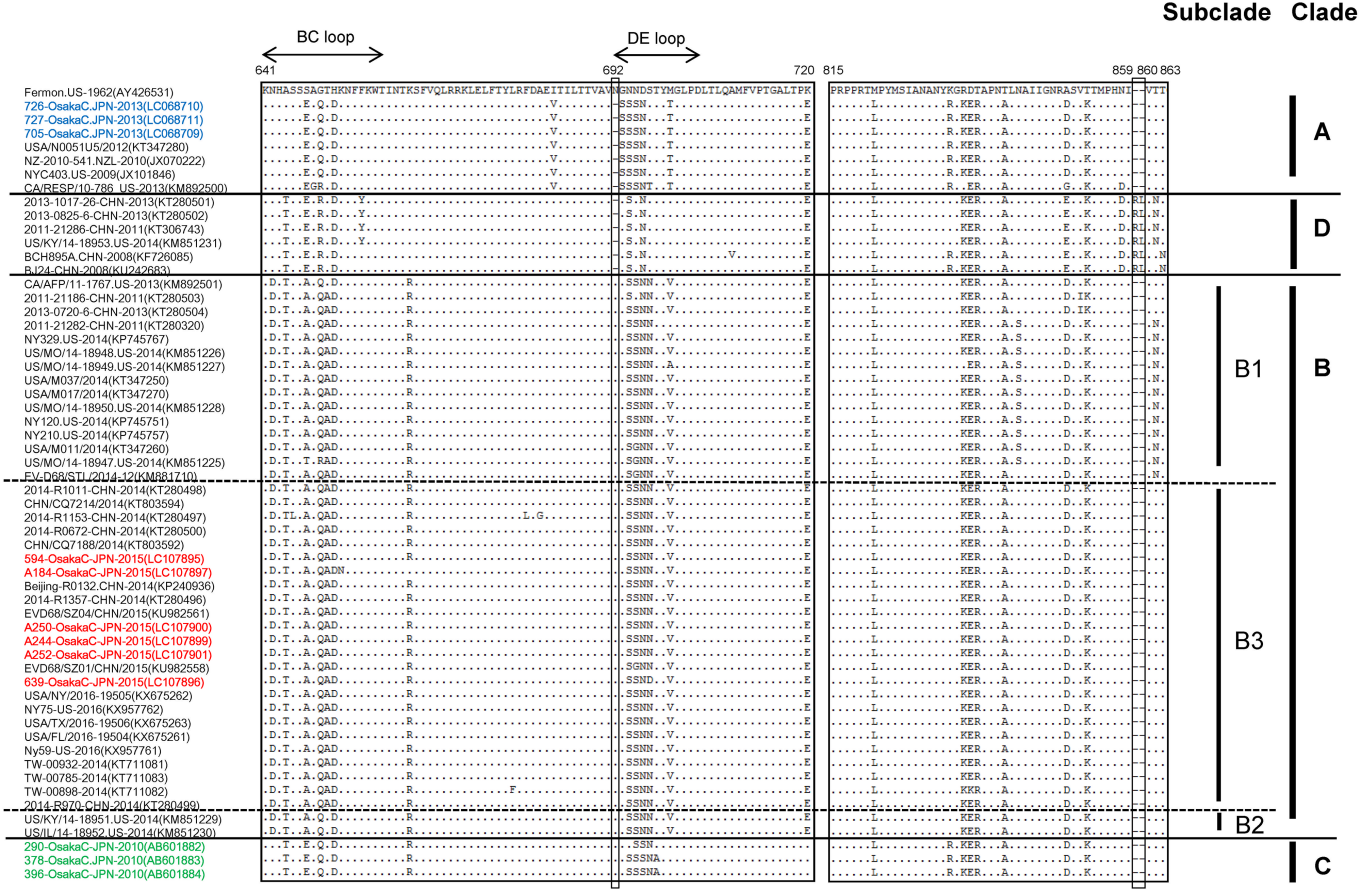
Comparison of VP1 region amino acid sequences between EV-D68 strains in distinct genetic clades. An alignment of partial amino acid sequences of the EV-D68 strains was analyzed. Strains in Fig 3 were used in this analysis.

To analyze VP1 in detail, amino acid sequences were aligned and examined for substitutions. BC and DE loops located on the surface of the VP1 protein play important roles in the antigenicity of HEV, and two specific amino acid substitutions (N642D, S647A) were observed in the BC loop in Clade B, and one amino acid deletion (N692) was observed in the DE loop in Clade A and Clade D. In addition, a two-amino-acid insertion (arginine—leucine) was also observed, but only at the 859–860 aa position in Clade D strains (Fig 4). Specific amino acid differences between clades or subclades in the same clade were also observed in the BC and DE loops, with substitutions such as N642D (Clade B), A644T (Clade B, C, D), S647E (Clade A, C, D), S647A (Clade B), T650A (excluding CA/AFP/11-1763 strains, Subclade B1 and B3), K662R (excluding A184-Osaka strain, Clade B, Clade C), and G693S and M700T (Clade A). The amino acid positions correspond to those of the Fermon strain.

### Partial deletion of the 5′ UTR

**Fig 5:**
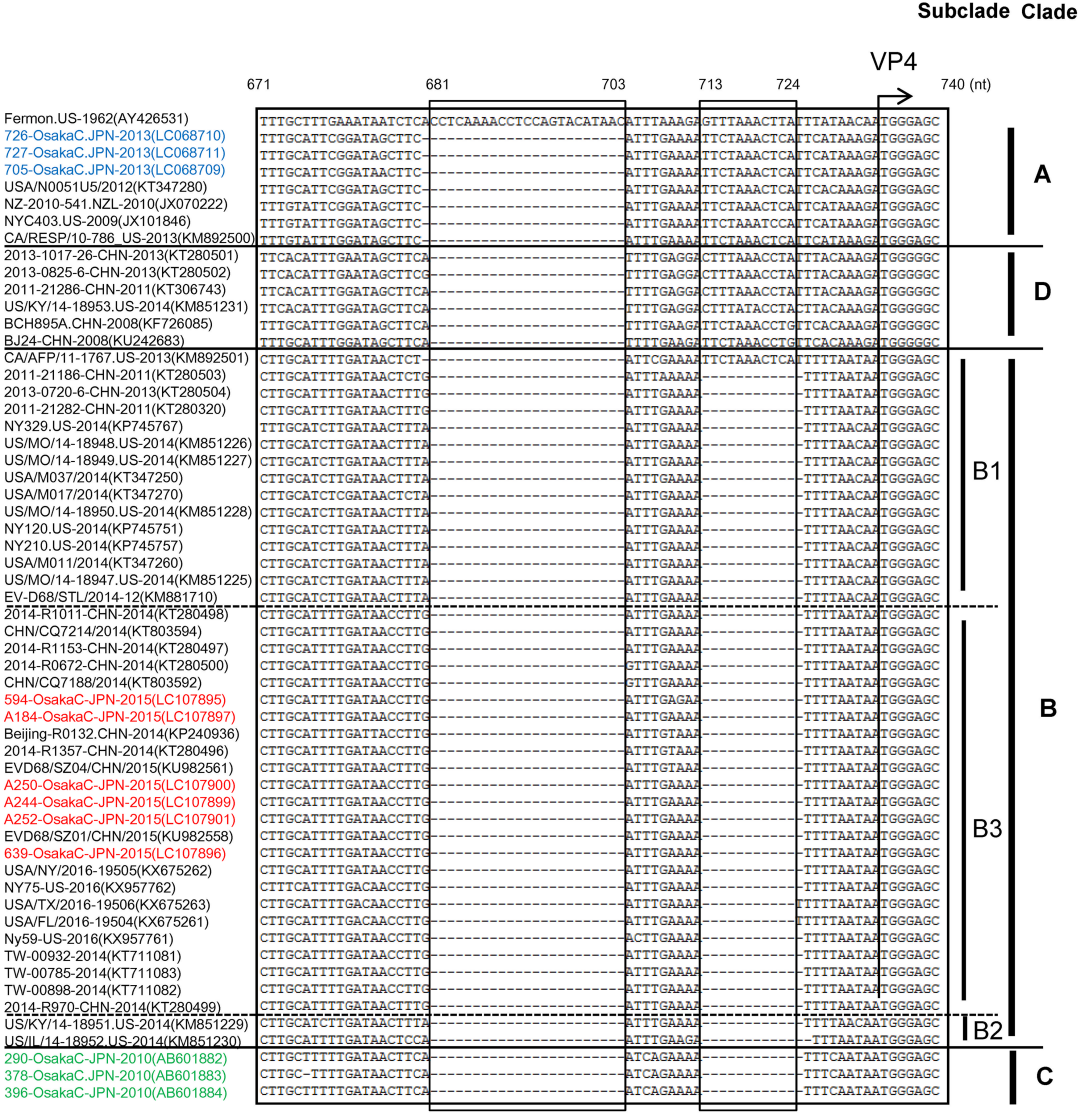
Alignment of nucleotide sequences of the 5′ UTR of strains in the three major genetic clades of EV-D68. Nucleotide sequences (nucleotide positions 501–1,000, corresponding to those of the Fermon strain) were aligned in MEGA 7.0. A partial sequence (nucleotide positions 671–740 of Fermon strain) is shown. Hyphens denote deleted nucleotides.

To analyze the 5′ UTR of the complete or near-complete genome strains, nucleotide positions 501–1,000, corresponding to those of the Fermon strain, were analyzed. All clades (A, B, C, D) of EV-D68 showed deletions at positions 681–703 nt compared to the Fermon strain. As for Clade A, one nucleotide at position 680 was also deleted. Additionally, nucleotides 713–724 were deleted from strains in Clades B and C, except for in strain CA/AFP/11-1767 in Clade B, which also showed one nucleotide deletion at position 680 (Fig 5).

## Discussion

This report is the first to describe longitudinal surveillance of EV-D68 in children with ARIs since the first epidemic in Japan in 2010. Of 18 EV-D68-positive patients, 15 (83.3%) were positive for EV-D68 alone. Therefore, EV-D68 is believed to be a cause of ARI in at least 15 of these cases. The specimens collected in 2013 were fewer than those collected in 2010–2012 or 2014–2015 because of fewer specimens collected from clinics and not because of a decreasing number of patients with ARI. The rates of EV-D68 positivity per year were 3.8%, 2.8%, and 1.9% in 2010, 2013, and 2015, respectively. The highest rates of EV-D68 were observed in August (15.4%) in 2010, October (25%) in 2013, and September (13.2%) in 2015. Taken together, these results indicate that EV-D68 epidemics in 2010, 2013, and 2015 in Osaka City might have occurred on a similar scale.

Re-emergences of EV-D68 in Osaka City occurred mainly from June to October (summer to autumn) in 2013 and 2015, similar to its first appearance in 2010 [17]. Other epidemiological data on EV-D68 in Japan, Yamagata Prefecture, showed an upsurge of EV-D68 between summer and autumn in 2005–2010 [12]. Based on the available data, summer through autumn is believed to be the EV-D68 season in Japan. Multi-year studies on EV-D68 suggest that the EV-D68 epidemic season differs by country, with peak seasons of August—January in China [23], July—December in Australia [24], October—December in Germany [25], and July—October in the Netherlands [26], and variable seasons in Thailand and Hong Kong [15, 27]. Data from a multi-year study conducted worldwide are needed to precisely define EV-D68 seasonality.

The phylogenetic analysis of complete or nearly complete genomes and VP1 sequences revealed that Osaka strains detected in 2010, 2013, and 2015 belonged to three genetic clades. Earlier reports describing receptor binding activity of EV-D68 strains in all genetic clades showed that distinct genetic clades have different antigenicities [28]. Therefore, differences in the prevalent genetic clade of EV-D68 each year in Osaka City might have contributed to its re-emergence in this limited geographic area. There were high similarities in VP1 nucleotide and amino acid sequences of Osaka strains detected in 2010, 2013, and 2015. However, lower similarities were observed for strains detected in 2010 vs. 2013, 2013 vs. 2015, and 2010 vs. 2015. This low similarity might be associated with differences in EV-D68 antigenicity. Lower herd immunity against strains in specific genetic clades of EV-D68 might have facilitated the rapid spread of a clade of EV-D68 that was not reported to be prevalent in the limited geographic area. Intriguingly, co-circulation of all three clades of EV-D68 was observed in Yamagata Prefecture, Japan, in the 2010 epidemic [12]. Based on these results, EV-D68 dynamics might differ in the same period in different parts of the country. One might infer that herd immunity against different clades of EV-D68 is associated with their prevalences in a limited geographic area.

Certain amino acid changes and deletions in the BC and DE loops of the VP1 protein were observed between the clades of EV-D68. Moreover, characteristic amino acid substitutions were observed in a comparison of strains in subclades of the same clade. Considering the effects of the BC and DE loops on the surface of the VP1 protein, EV-D68 strains belonging to different subclades of the same clade might have different antigenicities.

A partial deletion in the 5′ UTR was first reported based on our previous work [17]. Some characteristic partial deletions, relative to the prototype Fermon strain, were observed in the 5′ UTRs of EV-D68 strains in three clades. However, we did not analyze functional differences or the physiological importance of the deletion on EV-D68. To date, there has been no published report on the function of the EV-D68 5′ UTR. The 5′ UTR of an enterovirus contains an internal ribosome entry site (IRES) that is associated with translational efficiency and virulence [29–31]. Deletions in EV-D68 strains appeared to be in the region between the IRES and the open reading frame. Analysis of this deletion may yield important information about EV-D68. The 5′ UTR of EV-D68 strain CA/AFP/11-1767 in clade B showed a deletion similar to that of strains in Clade A. Du *et al*. analyzed 2014 North American EV-D68 outbreak using VP1, which revealed that CA/AFP/11-1767 strain was phylogenetically distant from other subclade B1 strains [32]. Their findings might partially explain one amino acid substitution (T650) in the BC loop of VP1 in strain CA/AFP/11-1767. However, the reason for the Clade A-like nucleotide deletion in the 5′UTR of strain CA/AFP/11-1767 remains unknown.

Tan *et al*. reported intersubclade recombination events in the VP2 region between EV-D68 strains in clades B1 and B2 [33]. However, no intersubclade recombination was observed in EV-D68 strains from Osaka City in 2015 (data not shown).

During the study period, four EV-D68-positive patients without signs of ARI were also found. These patients showed the following: AFP (*n* = 1 in 2013), fever (*n* = 1 in 2015), unknown fever (*n* = 1 in 2015), and rash (*n* = 1 in 2015) from respiratory specimens (Table 1). Although EV-D68 was detected in specimens from several individuals without ARI symptoms, whether EV-D68 was directly associated with specific clinical signs remains uncertain.

EV-D68 in cases of AFM were reported in the US from 2012 to 2014. All of these strains belonged to Subclade B1 [5]. In addition, EV-D68 in cases of AFP/AFM were reported in Japan in 2015 [34, 35]. However, no EV-D68-positive AFP/AFM cases were observed in Osaka City in 2015. Only one Clade A strain in 2013 was detected from a case of AFP (692-OsakaC-JPN-2013). A few reports describing EV-D68 from patients with AFP/AFM were published in 2010 and 2013 in Japan, but genetic clades of the infecting strains were not determined [34]. Two AFM cases in France and Norway were associated with Subclade B2 EV-D68 infection [6, 9], as were two neurological cases in Kenya with Subclade A1 [36] described in an earlier report [33]. Considering previous reports of neurological conditions associated with the detection of strains in various clades of EV-D68, only Subclade B1 strain was found predominant in AFM cases. It is not a major cause of AFM.

Overall, results of the analysis of complete or near-complete genome sequences suggest that the presence of strains in distinct lineages of EV-D68 in 2010, 2013, and 2015 might have contributed to the re-emergence of EV-D68 in this limited region because of lack of herd immunity. To analyze EV-D68 further, near-complete genome sequences of earlier reported strains from cases of ARI, AFM, encephalitis, central nervous system disorders, and other diseases must be determined. Recently, a novel genetic lineage of Subclade B3 was identified that now includes 2015 Osaka strains. Based on our phylogenetic analysis of VP1 (Fig 2), there are some strains (2013-0720-6-CHN, 2011-21186-CHN, CA/AFP/v12T00346-USA-2013, CU171-THA-2011, CU134-THA-2011) that do not clearly cluster in the currently recognized genetic subgroups in Clade B. Therefore, novel genetic subclades may be identified in the future.

To date, it is uncertain whether specific clades of EV-D68 are associated with specific clinical signs. Accumulating epidemiological information and analyzing sequences of EV-D68 strains are important to understanding the recent upsurge in cases of EV-D68 infection and might suggest associations between specific genetic clades and clinical signs.

## Supporting information

S1 Table Nine primer pairs used to amplify the EV-D68 genomes. (XLSX)
